# High expression of protein tyrosine kinase 7 in oral squamous cell carcinoma: Clinicopathological correlation and prognosis relevance

**DOI:** 10.1002/cre2.553

**Published:** 2022-03-08

**Authors:** Yujiro Kimura, Kunio Yoshizawa, Asami Hotta‐Osada, Akinori Moroi, Hiroki Ishii, Daiju Sakurai, Masao Saitoh, Naoki Oishi, Tetsuo Kondo, Koichiro Ueki

**Affiliations:** ^1^ Department of Oral and Maxillofacial Surgery, Interdisciplinary Graduate School of Medicine University of Yamanashi Chuo City Yamanashi Japan; ^2^ Department of Otolaryngology‐Head and Neck Surgery Interdisciplinary Graduate School of Medicine University of Yamanashi Chuo City Yamanashi Japan; ^3^ Department of Biological Chemistry, Center for Medical Education and Sciences, Interdisciplinary Graduate School of Medicine University of Yamanashi Chuo City Yamanashi Japan; ^4^ Department of Pathology, Interdisciplinary Graduate School of Medicine University of Yamanashi Chuo City Yamanashi Japan

**Keywords:** invasion, metastasis, oral squamous cell carcinoma, prognosis, protein tyrosine kinase 7

## Abstract

**Background:**

The purpose of this study was to evaluate the association between the　immunohistochemistry (IHC) of protein tyrosine kinase 7 (PTK7) expression and clinicopathological factors of oral squamous cell carcinoma (OSCC).

**Methods:**

Tissue specimens were obtained from 80 patients with primary OSCC. IHC scoring was conducted according to the rate of positive cell and staining intensity. We used the IHC score to classify the degree of PTK7 expression and evaluate clinicopathological factors and prognosis.

**Results:**

The number of the high expression group (IHC Score 2 or 3) was 45 cases and that of the low expression group (IHC Score 0 or 1) was 35 cases. A significant difference between high expression and low expression groups was found in the *N* category (*p* = .008), degree of differentiation (*p* < .001), and pattern of invasion (*p* < .001). In accordance with the exacerbation of OSCC with respect to three parameters (*N* category, degree of differentiation, and pattern of invasion), the ratio of high expression of PTK7 increased. The overall 5‐year survival rate was 59.3% in the high expression group and 87.3% in the low expression group (*p* < .05). The pathological prognostic signs affecting overall survival were evaluated by univariate analysis and multivariate analysis with Cox proportional hazards model and showed an association with lymph node metastasis and invasion patterns.

**Conclusion:**

This study suggests that a high IHC score of PTK7 is a potential biomarker for predicting potential metastasis.

## 1 INTRODUCTION

The sixth most common malignancy in the world is head and neck cancer, including oral squamous cell carcinoma (OSCC; Torre et al. (2015). It is estimated that in the United States, approximately 35,000 people will be diagnosed with OSCC and 7000 people will die from OSCC in 2020 (Siegel et al., 2020). Invasion and metastasis are the most important features of malignancy that affect prognosis. Various biomarkers, including clinicopathological factors, tumor sites, and TNM stage, have been used as prognostic parameters. However, these biomarkers cannot be used to predict invasion and occult metastasis (Rivera et al., 2017). Conversely, it has been considered that cancer cells located in the tumor invasive front of OSCC, are associated with regional metastasis and poorer survival (Almangush et al., 2017).

According to Jakobson's criteria (Jakobsson et al., 1973), the worst prognosis is Grade 4, but the classification of invasion patterns (YK classification) was proposed by Yamamoto et al. (1983) is characterized by the subdivision into Grade 4C (cord‐like type) and Grade 4D (diffuse type). The YK classification is used as a histopathological classification of the invasive capacity of OSCC and is often used to predict metastasis and prognosis by Japanese oral surgeons (Table 1) (Kato et al., 2008; Yamamoto et al. 1983). Understanding the invasion capacity of OSCC is important to provide appropriate treatment. In clinical practice, a biopsy is important in identifying the histology of OSCC tumors and confirming their stage. However, detecting the extent of highly invasive cancer using macroscopic findings from biopsy specimens is difficult (Seki et al., 2016). Therefore, finding new biomarkers that accurately identify highly invasive cancers and predict the metastasis and prognosis of OSCC are needed.

**Table 1:** Yamamoto–Kohama classification

Grade histological grading
YK1 well‐defined borderline
YK2 cords, less marked borderline
YK3 groups of cells, no distinct borderline
YK4C diffuse invasion, cord‐like type
YK4D diffuse invasion, widespread type
YK, Yamamoto–Kohama classification

Protein tyrosine kinase 7 (PTK7), also known as colorectal cancer kinase 4, is a receptor tyrosine pseudokinase lacking detectable catalytic activities (Sheetz et al., 2020). PTK7 is one of the coreceptors of the noncanonical Wnt/planar cell polarity signaling pathway (Katoh, 2005), and a previous report shows its strong involvement in the invasion of intrahepatic cholangiocarcinoma (Jin et al., 2014). PTK7 has been shown to be involved in vertebrate embryogenesis (Shnitsar & Borchers, 2008) and epithelial‐mesenchymal transition which plays an important role in cancer (Ataseven et al., 2013; Katoh 2005; Khramtsov et al., 2010; Geyer et al., 2011; Saitoh, 2015; Yoshizawa et al., 2013). PTK7 is highly expressed in colon cancer, lung cancer, gastric cancer, and intrahepatic cholangiocarcinoma, and is considered a potentially important prognostic marker (Mossie et al., 1995). However, few studies have evaluated PTK7 expression in OSCC. The aim of our study was to investigate PTK7 expression in OSCC with immunohistochemistry (IHC) and to correlate PTK7 expression with clinical data such as invasion and prognosis.

## 2 MATERIALS AND METHODS

### 2.1 Patients

Primary specimens of OSCC were collected from 80 patients who underwent surgical resection for the tongue, gingival, the floor of the mouth, and buccal mucosa cancers at the University of Yamanashi Hospital from January 2013 to January 2020. The patients (47 men and 33 women) ranged in age from 38 to 96 years (mean age, 66 years old ± 13.9). As described below, specimens were fixed in paraformaldehyde or formalin solution, embedded in paraffin, and stored at room temperature.

This study was approved by the Ethics Committee of the University of Yamanashi (Approval number 2328) and was conducted in compliance with all relevant guidelines and regulations. We obtained written informed consent from patients for the following: publication of the study, collection of clinical samples of patients, and use of the information.

### 2.2 IHC staining

Specimens were fixed in periodate–lysine–paraformaldehyde solution or 10% formalin solution and were then embedded in paraffin in preparation for serial Section (3 μm). Hematoxylin‐eosin (H–E) staining was used for histological examination. IHC staining was performed using the immunopolymer method after deparaffinization and rehydration. Endogenous peroxidase was removed in 3% hydrogen peroxide solution for 10 min, and sections were washed with phosphate‐buffered saline (PBS). We used 1% bovine serum albumin‐PBS as a blocking agent to prevent nonspecific binding. The sections were then incubated at room temperature with rabbit polyclonal PTK7 antibody (1:200; Proteintech, 17799‐1‐AP, Chicago, IL, USA) for 2 h and washed with PBS. Next, the secondary antibody (Histofine Simple Stain MAX‐PO (M), Nichirei Biosciences, Tokyo, Japan) was reacted at room temperature for 30 min and washed with PBS. IHC reaction was confirmed with 0.03% 3,3′‐diaminobenzidine tetrahydrochloride.

### 2.3 IHC analysis

IHC  analysis was performed quantitatively by two examiners blinded to the clinical and pathological parameters of the specimens, at ×100 magnification with a light microscope, BX50 (Olympus, Tokyo, Japan), after interexaminer calibration. Staining results were evaluated by IHC scoring. The following score was used in the assessment of PTK7 immunostaining: 0, no staining or unspecific staining of tumor cells; 1, weak and positive staining of less than 5% of tumor cells; 2, moderate and positive staining of more than 5%–50% of tumor cells; and 3, strong and positive staining of more than 50% of tumor cells. For analyzing PTK7 expression, the IHC scores were classified as follows: a score of 0–1 was considered the low expression, whereas a score of 2–3 was considered the high expression. Each case was observed three times.

### 2.4 RNA sequencing data and processing

Using The Cancer Genome Atlas (TCGA) data, we constructed a cohort of 85 patients with OSCC and retrieved all corresponding H–E images and RNA sequencing data. RNA HTSeq‐counts data were normalized and analyzed using the edgeR algorithm (Anders et al., 2013) in an R statistical environment with a false discovery rate adjustment of 0.05. H–E images were categorized by the pattern of invasion and evaluated in conjunction with the mRNA expression data. The Kruskal–Wallis test was used to compare PTK7 mRNA level distribution among normal adjacent, low‐grade tumors (YK1 and YK2), and high‐grade tumors (YK3, YK4C, and YK4D).

### 2.5 Statistical analysis

SPSS Statistics for Windows version 25.0 (IBM Corp., New York, USA) was used for data analysis. An analysis of the variance model was used to calculate the intraclass correlation coefficients (ICCs) to summarize the inter‐reader variability for OSCC samples examined with the PTK7 antibody. Interobserver reliability by ICC was interpreted as follows: <0.50, low; 0.50–0.75, moderate; 0.75–0.90, good; and 0.90–1, excellent (Koo & Li, 2016). The relationship between the IHC of PTK7 expression and clinicopathological features was analyzed for significance using Mann–Whitney *U* test. A Kaplan–Meier survival analysis was performed to evaluate the relationship between the IHC of PTK7 expression and overall survival time. The Cox proportional hazards model was used for multivariate analyses, and *p* < .05 was considered statistically significant. The optimal cut‐off point was estimated by the positive predictive value obtained from the receiver operating characteristic curve analysis. Multiple comparisons were performed using the two‐sample Mann–Whitney *U* test with the Holm–Bonferroni correction to identify significant differences between groups.

## 3 RESULTS

### 3.1 ICC

The ICCs for the assessment of the two reviewers in this study ranged from 0.827 to 0.925, indicating good interobserver reliability.

### 3.2 The relationship between the IHC of PTK7 expression and clinicopathological features of the patients with OSCC

**Figure 1:**
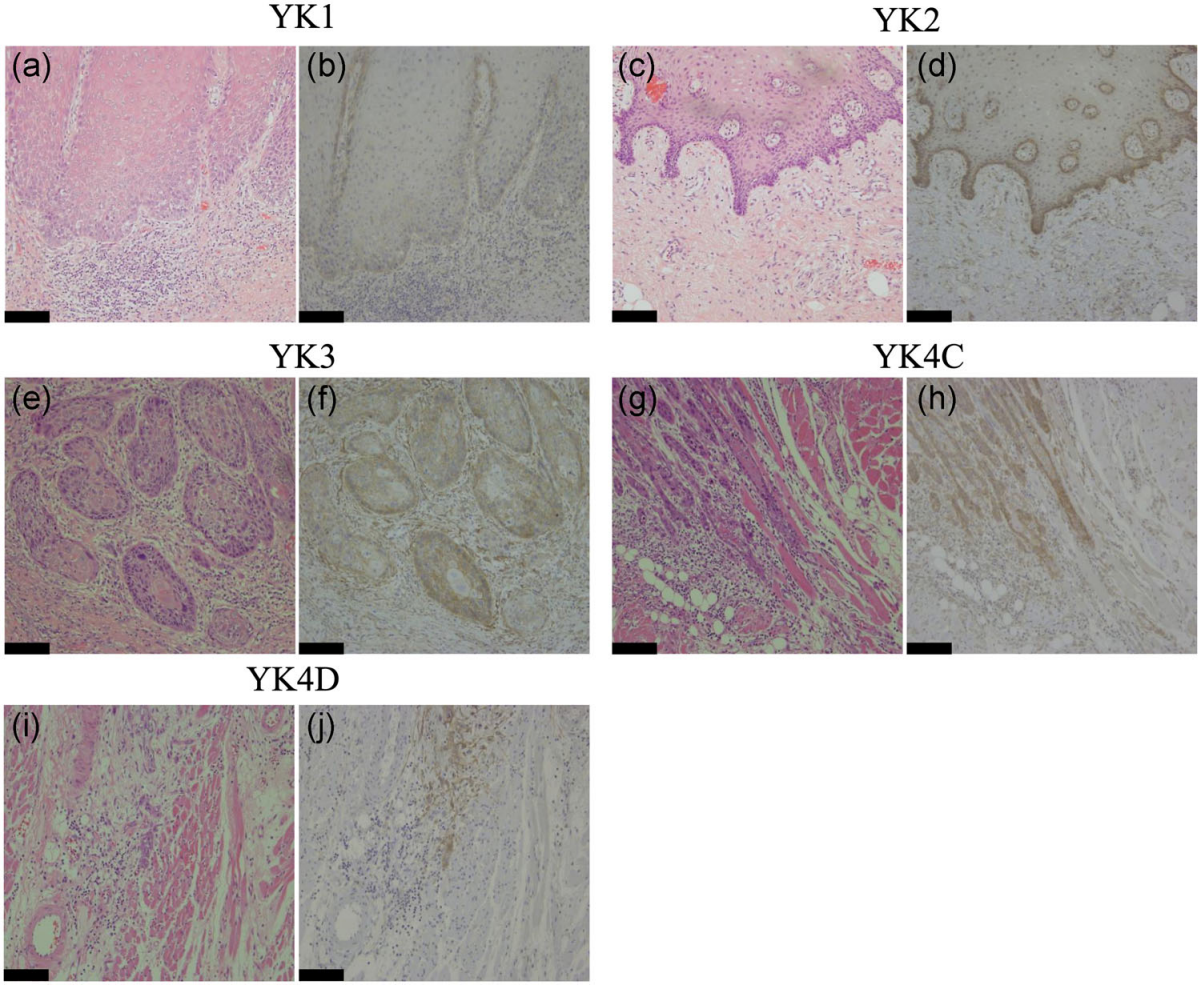
IHC reactivity for PTK7. Representative photos of the classified invasive pattern (H–E staining, and IHC PTK7 staining). IHC staining of PTK7 (b: weak expression; d, f: moderate expression; and h, j: strong expression). PTK7 immunoreactivity was found in the invasive front of OSCC. (a) YK‐1, H‐E staing. (b) YK‐1, PTK7 staining. (c) YK‐2, H‐E staing. (d) YK‐2, PTK7 staining. (e) YK‐3, H‐E staing. (f) YK‐3, PTK7 staining. (g) YK‐4C, H‐E staing. (h) YK‐4C, PTK7 staining. (i) YK‐4D, H‐E staing. (j) YK‐4D, PTK7 staining. Magnification: Original ×100. Scale bar = 100 μm. H–E, hematoxylin–eosin; IHC, immunohistochemistry; OSCC, oral squamous cell carcinoma

IHC revealed markedly increased PTK7 expression in OSCC tumor tissue compared with that in the adjacent normal tissue, and strong staining was predominantly observed in the basal layer and invasive front of tumor tissue (Figure 1). PTK7 localized to the cytoplasm and cytomembrane in cancer cells. We summarized the relationships between clinicopathological parameters and the IHC of PTK7 expression in Table 2. In total, 45 patients (56.3%) had high PTK7 expression (IHC Score 2, 17 patients; IHC Score 3, 28 patients), whereas 35 patients (43.7%) had low PTK7 expression (IHC Score 0, 18 patients; IHC Score 1, 17 patients). A significant difference between high expression and low expression groups was found in *N* category (*p* = .008), degree of differentiation (*p* < .001), and pattern of invasion (*p* < .001). In accordance with the exacerbation of OSCC with respect to three parameters (*N* category, degree of differentiation, and pattern of invasion), the ratio of high expression of PTK7 increased (Table 2). PTK7 immunostaining was mainly observed in the basal layer in YK1 and YK2 of YK classification as well as in whole cells of the infiltrating protrusions in YK3, YK4C, and YK4D grade (Figure 1). A significant positive correlation was found between PTK7 and the pattern of invasion (*p* < .001). The number of patients with high PTK7‐expression by YK class were as follows: YK1, 5 (33.3%); YK2, 6 (42.9%); YK3, 15 (48.3%); YK4C, 15 (88.2%); and YK4D, 5 (100%). No significant difference was found between the IHC of PTK7 expression level and each of age, gender, postoperative radiation therapy, and cancer recurrence (data not shown).

**Table 2:** Association between PTK7 expression and clinicopathological parameters in OSCC

Parameter		High expression no. (%)	Low expression no. (%)	Total	*p* value
Age (years)					.269
65≥		24 (51.0)	23 (49.0)	47	
65<		21 (63.6)	12 (36.3)	33	
Gender					.481
Male		28 (59.5)	19 (40.5)	47	
Female		17 (51.5)	16 (48.5)	33	
Tumor site					.365
Tongue		34 (60.7)	22 (39.3)	56	
Gingiva		3 (27.3)	8 (72.7)	11	
Oral floor		2 (50.0)	2 (50.0)	4	
Buccal		4 (80.0)	1 (20.0)	5	
Others		2 (50.0)	2 (50.0)	4	
T category					.098
T1		15 (42.9)	20 (57.1)	35	
T2		18 (66.7)	9 (33.3)	27	
T3		5 (71.4)	2 (28.6)	7	
T4		7 (63.6)	4 (36.3)	11	
N category					**.008**
N0		30 (48.4)	32 (51.6)	62	
N1		6 (75.0)	2 (25.0)	8	
N2		9 (90.0)	1 (10.0)	10	
Cell differention					**<.001**
Well		20 (40.8)	29 (59.2)	49	
Moderate		16 (72.7)	6 (27.3)	22	
Poor		9 (100)	0 (0)	9	
Pattern of invasion					**<.001**
YK1		5 (33.3)	10 (66.7)	15	
YK2		6 (42.9)	8 (57.1)	14	
YK3		14 (48.3)	15 (51.7)	29	
YK4C		15 (88.2)	2 (11.8)	17	
YK4D		5 (100)	0 (0)	5	

*Note*: *p* Values in bold print indicate statistical significance. *p* Values are analyzed via the Mann–Whitney U test.

Abbreviations: OSCC, oral squamous cell carcinoma; YK, Yamamoto–Kohama classification.

### 3.3 Summary of survival analysis using clinical samples

**Figure 2:**
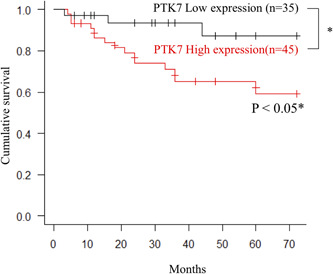
Kaplan–Meier survival analysis based on the PTK7 expression. Kaplan–Meier plots demonstrate the correlation between PTK7 IHC expression and patients in OSCC (*n* = 80). Redline, high expression (*n* = 45); black line, low expression (*n* = 35). *, a statistically significant difference. IHC, immunohistochemistry; OSCC, oral squamous cell carcinoma

The Kaplan–Meier analysis revealed that the high IHC of PTK7 expression was correlated with poor overall survival (Figure 2). The overall 5‐year survival rate was 59.3% in the high expression group, versus 87.3% in the low expression group (*p* < .05). Univariate analysis showed particularly strong associations in the T category, N category, the pattern of invasion, cell differentiation, and the IHC score (Table 3). Therefore, we decided to perform a multivariate analysis focusing on all five categories and found differences in lymph node metastasis and invasion patterns but no differences in the high IHC of PTK7 expression level (Table 4).

**Table 3:** Univariate Cox regression models for estimating the overall survival

		Cox regression analysis		
Variables	Clinical groups	HR	95% CI	*p* value
Univariate analysis				
Age	≥65/<65	1.899	0.6225–5.793	.2597
Gender	Male/female	1.233	0.486–3.127	.6596
T category	1/2,3, 4	15.53	2.059–117.1	**<.01**
N category	N0/N+	14.64	4.806–44.62	**<.001**
Pattern of invasion	1–3/4C–4D	9.549	3.383–26.95	**<.001**
Cell differentiation	Well/moderate–poor	9.095	2.628–31.47	**<.001**
IHC score	Low/high expression	3.929	1.135–13.6	**<.05**

*Note*: *p* Values in bold print indicate statistical significance.

Abbreviations: CI, confidence interval; HR, hazard ratio; IHC, immunohistochemistry.

**Table 4:** Multivariate Cox regression models for estimating the overall survival

		Cox regression analysis		
Variables	Clinical groups	HR	95% CI	*p* value
T category	1/2,3, 4	6.746	0.8514–53.46	.0706
N category	N0/N+	7.09	2.056–24.44	**<.001**
Pattern of invasion	1–3/4C–4D	6.149	1.245–30.37	**<.05**
Cell differentiation	Well/moderate–poor	0.5889	0.2162–1.604	.3004
IHC score	Low/high expression	1.037	0.2213–4.858	.9634

*Note*: *p* Values in bold print indicate statistical significance.

Abbreviations: CI, confidence interval; HR, hazard ratio; IHC, immunohistochemistry.

### 3.4 TCGA data analysis

A significant difference in PTK7 mRNA relative to the normal control expression was observed in YK‐3, YK‐4C, and YK‐4D (*p* < .001, Figure S1).

## 4 DISCUSSION

This study revealed that the invasiveness of OSCC increased with increasing PTK7 expression. The multivariate Cox regression model identified significant differences in the N category and the pattern of invasion according to PTK7 expression.

Periostin is a matricellular protein secreted by cancer‐associated fibroblasts (Xu et al., 2016) and the interaction between periostin and PTK7 regulates the canonical Wnt signaling pathway (Yu et al., 2018). Many reports suggest that the canonical Wnt signaling pathway contributes to the activation of carcinogenesis (Polakis 2007; Sheikh et al., 2014; Rosenbluh et al., 2014). On the other hand, in the noncanonical Wnt signaling pathway, PTK7 regulates a variety of processes in embryonic development (Katoh, 2005). The correlation between PTK7 expression patterns and cancerization is controversial for cancer lesions in various organs. High PTK7 expression has been found in various cancers, including gastric cancer (Lin et al., 2012) and intrahepatic cholangiocarcinoma (Jin et al., 2014). However, PTK7 is downregulated in lung squamous cell carcinoma (Kim et al., 2014) and ovarian carcinoma (Wang et al., 2014). In addition, the correlation between PTK7 expression and cancer invasion/metastasis and prognosis has been found to vary by organ and tumor types (Berger et al. 2017; Dunn & Tolwinski, 2016). For example, Jin et al. (2014) reported that high PTK7 expression at the mRNA level is associated with invasion and poor prognosis in intrahepatic cholangiocarcinoma. In head and neck cancer, PTK7 mRNA expression was higher in cancer cells than in normal cells, using TCGA data (Yu et al., 2018). By contrast, Lin et al. (2012) reported that high PTK7 expression in gastric cancer as assessed by IHC analysis was a positive prognostic factor for favorable overall survival and disease‐free survival. Although PTK7 expression was negatively correlated with prognosis including *N* category and the pattern of invasion in this study, few reports have described the relationship between PTK7 expression and the invasiveness of OSCC. Therefore, we investigated whether PTK7 expression changed according to the degree of invasiveness of OSCC using TCGA and mRNA data. We found that the invasiveness of OSCC increased with increasing PTK7 expression in the TCGA database. However, many cases in the TCGA database did not contain clinical information such as survival time, and thus, we were unable to examine the relationship between PTK7 expression and survival. In the future, it will be necessary to incorporate more clinical information from the TCGA database and conduct detailed immunohistochemical analyses, including analyses of the associations between the localization of positive staining and clinicopathological factors.

Due to the limitation of using the TCGA database to confirm PTK7 mRNA expression combined with sufficient clinical information, we need to investigate mRNA expression in clinical samples in our laboratory. Based on previous reports, it is known that PTK7 interacts with periostin, participates in Wnt signaling, and contributes to malignant transformation, and thus, the mechanism should also be investigated using a molecular biological approach. In conclusion, we found that high mRNA expression of PTK7 predicts invasion and potential metastasis, thereby suggesting that PTK7 is a potential prognostic biomarker for OSCC.

## CONFLICTS OF INTEREST

The authors disclosed no conflicts of interest.

## AUTHORS' CONTRIBUTIONS

Yujiro Kimura, Kunio Yoshizawa, Naoki Oishi, and Tetsuo Kondo participated in the design of the study. Kunio Yoshizawa, Asami Hotta‐Osada, and Akinori Moroi contributed to the investigation. Hiroki Ishii and Daiju Sakurai contributed to the resources, which is the pathological sections of otorhinolaryngology. Yujiro Kimura and Kunio Yoshizawa participated in the writing of the manuscript and data analysis. Kunio Yoshizawa, Koichiro Ueki, and Masao Saitoh contributed to the editing. All authors read and approved the final manuscript.

## Supporting information


Figure S1.

## Data Availability

The data that support the findings of this study are available from the corresponding author (yoshizawak@yamanashi.ac.jp) upon reasonable request.
