# Supplementary figures and images for: High expression of protein tyrosine kinase 7 in oral squamous cell carcinoma: Clinicopathological correlation and prognosis relevance
Source: Clin Exp Dent Res. 2022 Mar 8;8(2):506–12. doi: 10.1002/cre2.553 (PMC9033537; doi:10.1002/cre2.553)

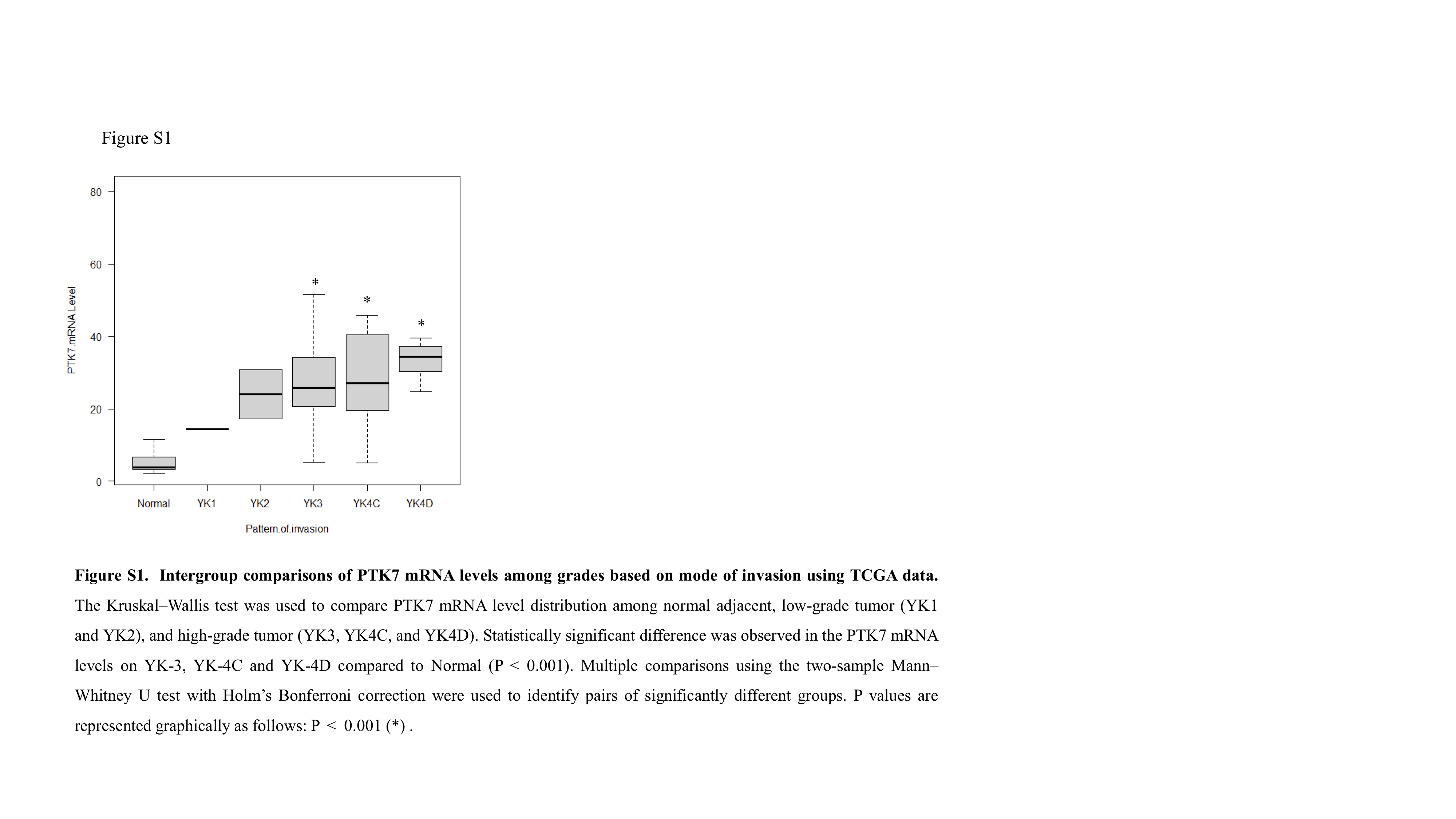

Supplement: Supplementary file 1 — Figure S1. [file CRE2-8-506-s001.tif]
